# Gut microbiota and their metabolites in the progression of non-alcoholic fatty liver disease

**DOI:** 10.20517/2394-5079.2020.134

**Published:** 2021-01-13

**Authors:** Jin Zhou, Madhulika Tripathi, Rohit A. Sinha, Brijesh Kumar Singh, Paul M. Yen

**Affiliations:** 1 Program of Cardiovascular and Metabolic Disorders, Duke-NUS Medical School, Singapore 169857, Singapore; 2 Department of Endocrinology, Sanjay Gandhi Postgraduate Institute of Medical Sciences, Lucknow 226014, India; 3 Duke Molecular Physiology Institute, Durham, NC 27701, USA; 4 Duke University School of Medicine, Durham, NC 27710, USA

**Keywords:** Non-alcoholic fatty liver disease, gut microbiome, gut microbiota metabolites

## Abstract

Non-alcoholic fatty liver disease (NAFLD) is the most prevalent liver disorder worldwide. It comprises a spectrum of conditions that range from steatosis to non-alcoholic steatohepatitis, with progression to cirrhosis and hepatocellular carcinoma. Currently, there is no FDA-approved pharmacological treatment for NAFLD. The pathogenesis of NAFLD involves genetic and environmental/host factors, including those that cause changes in intestinal microbiota and their metabolites. In this review, we discuss recent findings on the relationship(s) of microbiota signature with severity of NAFLD and the role(s) microbial metabolites in NAFLD progression. We discuss how metabolites may affect NAFLD progression and their potential to serve as biomarkers for NAFLD diagnosis or therapeutic targets for disease management.

## Introduction

Non-alcoholic fatty liver disease (NAFLD) is one of the most prevalent chronic liver diseases worldwide^[1]^. NAFLD is characterized by fat accumulation and defined by the presence of steatosis in > 5% of hepatocytes according to histological analysis without any history of significant alcohol consumption or viral hepatitis^[2]^. NAFLD includes two pathological conditions: non-alcoholic fatty liver (NAFL) and non-alcoholic steatohepatitis (NASH). Among the two conditions, NASH represents a more severe form and is characterized by progressive inflammation, hepatocyte death, and fibrosis^[2]^. Recently, it has been proposed that NAFLD be renamed as metabolic dysfunction associated with fatty liver disease (MAFLD) to highlight its relationship to metabolic conditions such as obesity, diabetes, hypertriglyceridemia, hypercholesterolemia, and atherosclerosis. If universally adopted, this new nomenclature and definition will have a great impact on clinical practice^[3,4]^, particularly with respect to the diagnosis of patients and endpoints for clinical trials^[5]^. However, in alignment with previously published literature, the current review continues to use the term “NAFLD”.

NAFLD is a chronic and complex disease that may be attributed to a combination of genetic and environmental factors. NAFLD is highly associated with obesity, type 2 diabetes mellitus (T2DM), and dyslipidemia^[1]^. “Multiple parallel hits” have been postulated to explain the complex molecular pathogenesis underlying the evolution from NAFLD to NASH^[6]^. Alteration of gut microbiota is thought to be one of the hits that contributes to pathogenesis of NAFLD. Indeed, the gut microbiome affects the lipid metabolism, apoptosis, inflammation, and fibrosis during NAFLD progression. Therefore, the aim of this review is to highlight the molecular mechanism(s) for microbiota induction of NAFLD progression and the current strategies to manage NAFLD by manipulating microbiota or its metabolites.

## Gut Microbiota in NAFLD Progression

The intestine harbors a large quantity of microorganisms, mostly comprising of bacteria, that are collectively called gut microbiota. Their total number in the body is estimated to be approximately 40 trillion, which is close to the total number of human cells in a person, and their total mass is approximately 0.2 kg^[7]^. Bacteria belong to specific taxonomic groups, comprising phyla, classes, orders, families, genera, and species. More than 90% of the microbiota in the gut microbiome belong to two phyla, Firmicutes and Bacteroidetes^[8]^. Recent advances in molecular biology techniques, particularly in sequencing and bioinformatic analysis, enable detailed characterization of the composition and diversity of the gut microbiome. Microbial diversity begins within the first few hours after birth and is shaped during childhood and adolescence when the diet becomes more diverse and the immune system matures. In contrast, the composition of adult microbiota in the gut remains relatively stable^[9]^. The gut microbiota also can change rapidly in response to environmental factors such as alterations in lifestyle, medications, or diet.

As a co-evolved system, the microbiota function almost as a “metabolic organ” that impacts nutrition and energy homeostasis. It can also be affected by, as well as contribute to, the progression of metabolic diseases, including NAFLD. In this connection, experimental evidence from animals demonstrates a direct role for gut microbiota in the development of NAFLD. Inoculation of fermentative microbial strains *B. thetaiotaiomicron* and *M. smithii* into adult germ-free (GF) C57BL/6 mice increased energy harvest from the diet as well as host adiposity regardless of reduced food intake. These microbial strains promote absorption of monosaccharides from the gut lumen, which results in induction of *de novo* hepatic lipogenesis^[10]^. Furthermore, germ-free mice were protected against Western-style diet-induced obesity^[11]^. A subsequent study further demonstrated that the differences in microbiota composition influenced the development of hepatosteatosis by high-fat diet (HFD)^[12]^. Most wild-type mice fed with HFD developed insulin resistance and systemic inflammation (responders); however, some mice remained insulin-sensitive and developed lower levels of systemic inflammation (non-responders). GF mice were then inoculated with gut microbiota from either responders or non-responders and then fed on HFD. Interestingly, non-responder recipient mice had less hepatosteatosis and insulin resistance than responder recipient mice. Further analysis revealed that the responder mice had increased numbers of Firmicutes phylum, *Barnesiella* and *Roseburia* genera, *Lachnospiraceae bacterium* 609, and *Barnesiella intestinihominis* ^[13]^.

## Dysbiosis in NAFLD Patients

Alterations in gut microbiota composition and function have negative impacts on the host (dysbiosis) and play causal roles in the development of NAFLD. In a study in which gut microbiota collected from obese donors before and after weight loss were transplanted into GF mice, the mice that received gut microbiota before weight loss had higher levels of hepatic triglyceride and cholesterol than mice that received post-weight loss gut microbiota^[14]^. The gut microbiota in infants of obese mothers also increased inflammation and susceptibility to NAFLD^[15]^.

Several studies have analyzed the gut microbiota composition in NAFLD patients and control subjects^[16]^. Increased Proteobacteria at the phylum level was seen in NAFLD patients compared to control subjects^[17–21]^. Increased Enterobacteriaceae^[18,21]^, decreased Rikenellaceae^[19,22]^ and Rumminoccaceae^[18–21]^ at the family level were found in NAFLD patients. Increased Escherichia^[17,21]^ and Dorea^[19,23]^ and decreased Anaerosporobacter^[22,24]^, Coprococcus^[17,21,22]^, Eubacterium^[17,21]^, Faecalibacterium^[21,24]^, and Prevotella^[17,25]^ were observed at the genera level in NAFLD patients. Microbial signatures of NAFLD-related fibrosis or cirrhosis also have been investigated^[20,26–28]^. Patients with advanced fibrosis displayed increased Gram-negative microbes and proteobacteria, and decreased Firmicutes at the phylum level. Escherichia coli and Bacteroides vulgatus were increased and Eubacterium rectale was decreased at the species level^[20]^. Species within the Enterobacteriaceae family^[27]^ and the genera *Streptococcus* ^[27,28]^ and *Gallibacterium* ^[28]^ were most enriched in NAFLD-cirrhosis patients. Increased *Prevotella copri* was associated more severe fibrosis in children with NASH^[26]^.

However, it is worth noting that discrepant results have been found across studies^[16,24,29–31]^. Variables such as exclusion criteria of subjects, ethnicity, and methods (16S-pyrosequencing vs. shotgun metagenomic sequencing) may account for these differences^[32]^. Nonetheless, several models that combined microbial species and a few clinical parameters were able to predict advanced fibrosis in patients with NAFLD^[20]^; thus, the detection of microbial markers may serve as non-invasive predictors of fibrosis [Table 1].

## Endotoxins and Gut Microbiota Metabolites in NAFLD

### Endotoxin effects on intestinal permeability

The majority of the gut microbiota species are colonized in the large intestine^[33]^; however, clinical evidence suggests that NASH patients have a higher prevalence of small intestinal bacterial overgrowth (SIBO)^[34,35]^. SIBO is usually defined as the presence of > 10^5^ colony-forming units (CFU)/mL in duodenal aspirate cultures^[36]^. The presence of SIBO in NAFLD patients is normally determined by a breath test to measure the concentration of hydrogen and/or methane in the exhaled air which is produced by intestinal bacterial metabolism^[37]^. Ghoshal *et al*.^[38]^ used the quantitative jejunal aspirate culture and the glucose hydrogen breath test to detect low-grade SIBO in NASH patients. In this connection, Fei *et al.* ^[39]^ demonstrated that three endotoxin producing strains, *Enterobacter cloacae* B29, *Escherichia coli* PY102, and *Klebsiella pneumoniae* A7, overgrew in the gut of two morbidly obese volunteers. When introduced to GF mice fed on HFD, *E. cloacae* B29 caused NAFLD, whereas HFD alone did not induce the disease. Furthermore, *E. cloacae* B29 induced NAFLD in a TLR4-dependent manner.

One proposed mechanism that links dysbiosis and SIBO with NAFLD is intestinal permeability and the increased circulating endotoxins levels that trigger hepatic inflammatory or fibrotic response. Intestinal permeability can be measured by lactulose/mannitol test or the urinary excretion of Cr-ethylene diamine tetraacetate^[35,40,41]^, and increased intestinal permeability has been demonstrated in NAFLD patients^[42,43]^. An animal study showed that one week of HFD feeding was able to induce enough dysbiosis to cause significant gut vascular barrier damage for bacterial translocation^[43]^. Several studies have also suggested that endotoxins such as Lipopolysaccharides (LPS) increased intestinal permeability through activation of TLR4/MyD88 pathways^[44,45]^. Additionally, endotoxin levels were higher in patients with steatosis^[46,47]^ and were further increased after the transition from hepatosteatosis to NASH^[41,48,49]^.

### Endotoxin effects on hepatic inflammation and apoptosis

Animal studies have also demonstrated a causative role of LPS in metabolic syndrome and hepatic steatosis. Continuous subcutaneous infusion of low-dose LPS results in increased hepatic triglyceride in mice that were fed with standard chow diet^[50]^. Intraperitoneal injection of LPS exacerbated liver injury in mice fed a methionine- and choline-deficient diet (MCD)^[51]^. These studies indicated that the liver is one of the main targets of LPS, and, when the latter was bound to LPS-binding protein (LBP)-CD14 complex, it activated hepatic TLR4 to trigger the inflammatory cascade and promote NAFLD progression^[52,53]^. LPS also stimulates hepatocyte apoptosis, which is prominent in human NASH^[54]^ and may be another trigger for liver fibrosis^[55]^. Zhao *et al*.^[56]^ demonstrated that disruption of the AMPK-caspase 6 axis caused liver damage in NASH. When AMPK signaling was impaired, caspase 6 phosphorylation was decreased and thereby activated to generate a feed-forward loop that promotes apoptosis. In mice fed MCD diet, injection of LPS intraperitoneally further increased the production of TNFα as well as caused cell death^[51]^; thus, endotoxins have effects that regulate both inflammation and cell death.

**Figure 1:**
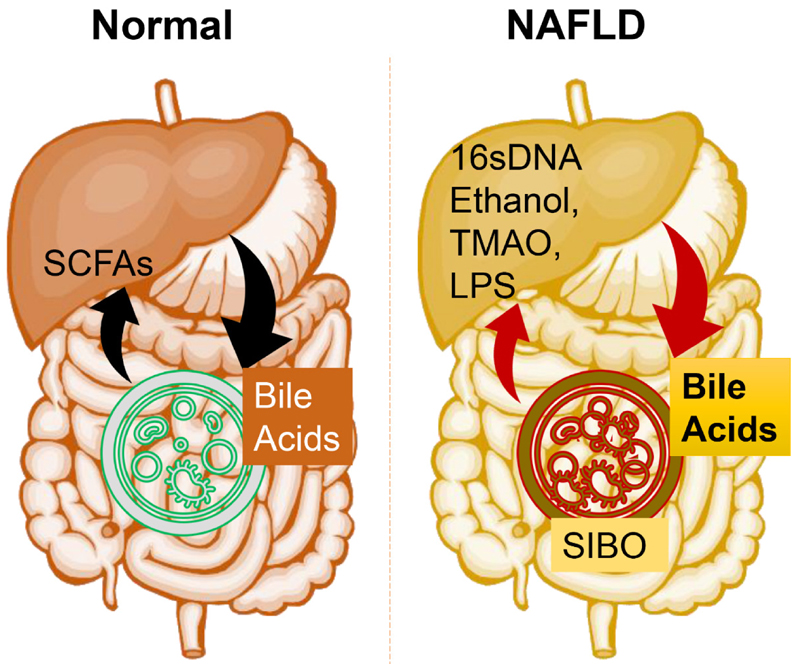
Gut microbial homeostatic balance is maintained under normal conditions. Gut microbiota produced SCFAs, namely acetate, butyrate, and propionate, influence hepatic metabolism by changing epigenetics/gene expression or directly via energy metabolism. Liver produced bile acids (such as cholic acids) are also processed by gut microbiota and released systemically. There is substantial microbial dysbiosis during NAFLD that causes SIBO of Gram-negative bacteria while reducing the overall microbial diversity. This bacterial overgrowth leads to produce proinflammatory molecules such as LPS, ethanol, TMAO, and bacterial 16sDNA. These proinflammatory molecules worsen the liver inflammation and fibrosis and potentially accelerate NAFLD progression. SCFAs: short chain fatty acids; SIBO: small intestinal bacterial overgrowth; LPS: Lipopolysaccharides; TMAO: trimethylamine N-oxide; NAFLD: Non-alcoholic fatty liver disease

Besides endotoxins, other gut bioactive metabolites including bacterial DNA and peptidoglycans may also contribute to NAFLD progression [Figure 1]. Mitochondrial DNA strongly activated TLR9 to drive NAFLD progression^[57–59]^. Substituents of peptidoglycans (PGNs), such as meso-diaminopimelic acid PGN (meso-DAP PGN) and muramyl dipeptide PGN (MDP PGN), generated proinflammatory cytokines through nuclear factor-κB (NF-κB)/mitogen-activated protein kinase (MAPK)-dependent activation of NOD1 (Nucleotide Binding Oligomerization Domain Containing 1) and NOD2 (Nucleotide Binding Oligomerization Domain Containing 2)^[60]^.

### Altered bile acid metabolism in NASH and its effects on hepatic inflammation and apoptosis

Bile acids are synthesized from cholesterol and in the liver. The primary bile acid species, cholic acid (CA) and chenodeoxycholic acid (CDCA), are further conjugated with glycine or taurine and stored in the gallbladder before release into the intestine after a meal^[61]^. In the intestine, bile acids promote the absorption of dietary fat, cholesterol, and fat-soluble vitamins. The primary bile acid species are deconjugated and dehydroxylated by intestinal microbiota to more hydrophobic secondary bile acid species, namely deoxycholic and lithocholic acid^[62]^, which are passively reabsorbed in the distal ileum and returned to the liver via the portal vein^[63]^. Some bacterial species in the colon, including Bacteroides, Clostridium, and Escherichia, can deconjugate and/or dehydroxylate bile acids, which may result in changes in the circulating unconjugated bile acids^[64]^.

Several studies revealed that NASH patients have higher serum levels of bile acids, including secondary bile acids, than healthy subjects^[64,65]^. A bile acid synthesis intermediate and marker for *de novo* bile acid synthesis, 7α-hydroxy-4-cholesten-3-one (C4), was higher in the serum in NASH patients, as well as associated with changes in fecal microbiota^[66,67]^. Some studies have suggested that there may be specific bile acid profile(s) associated with NASH. Yara *et al.* ^[68]^ analyzed the serum bile acids profile of NASH patients and healthy subjects and found that ratios of primary bile acid to secondary bile acids, taurine-conjugated bile acids to glycine-conjugated bile acids, unconjugated bile acids to total bile acids, and secondary bile acids to total bile acids were decreased in NASH patients. Chen *et al*.^[69]^ showed that increased ratios of circulating conjugated chenodeoxycholic acids (CDCAs) to muricholic acids in NASH patients were positively associated with the histological severity of NASH and fibrosis. Bile acids can also function as signaling molecules to modulate various biological processes [Table 2]. Regulatory actions of bile acids are mainly mediated through its receptors, including G-protein-coupled bile acid receptor 1 (TGR5, encoded by GPBAR1 gene) and farnesoid X receptor (FXR)^[70,71]^. Different bile acids have variable abilities to activate these receptors^[72]^.

Secondary bile acids taurolithocholic acid and lithocholic acid are more potent than primary bile acids in activating TGR5^[73]^. TGR5 has been found to be ubiquitously expressed in the human body with high levels of TGR5 mRNA detected in metabolically active organs such as small intestine, stomach, and liver. Activating TGR5 increased intestinal glucagon-like peptide-1 (GLP-1) release to increase glucose tolerance in obese mice^[74]^. TGR5 also was expressed in monocytes, macrophages, and Kupffer cells, and modulated immune response^[73,75]^. Indeed, in isolated Kupffer cells, bile acids activated TGR5 and inhibited LPS-induced cytokine expression in a cAMP-dependent manner^[75]^.

Besides their effects on inflammation, exposure to excessive bile acids in the liver can induce hepatocyte apoptosis. Bile acids activated JNK pathway and sensitized liver cells to apoptosis mediated by TNF-related apoptosis-inducing ligand (TRAIL)^[76,77]^. Bile acids also increased the aggregation of Fas receptor on the plasma membrane and initiated Fas receptor-mediated apoptosis^[78,79]^. Interestingly, bile acid-mediated apoptosis and synthesis were reduced by overexpression of an autocrine hepatic growth factor, Augmenter of Liver Regeneration (ALR)^[80]^.

### FXR effects on the liver in NASH

FXR is highly expressed in the intestine and liver, and it can be activated by free and conjugated primary bile acids. In the small intestine, FXR activation induces the fibroblast growth factor FGF19 in human or FGF15 in mouse, which binds to FGF receptor 4 (FGFR4) in the liver to repress bile acid synthesis^[81]^. Activation of hepatic FXR also regulates *de novo* lipogenesis, VLDL secretion, and gluconeogenesis in the liver^[82]^.

Synthetic FXR agonists haven been developed for treatment of NASH. Semisynthetic bile acid derivative obeticholic acid (OCA, 6α-ethyl-chenodeoxycholic acid), previously known as INT-747, is an agonist of FXR that is 100 times more potent than CDCAs. When type 2 diabetes mellitus and NASH were treated with 25 mg OCA for six weeks, patients had improved insulin sensitivity and decreased markers for fibrosis. These changes were associated with increased FGF19 as well as decreased levels of C4 and endogenous bile acids, consistent with FXR activation^[83]^. In a double-blind, placebo-controlled phase II trial for patients with NASH, when NASH patients were treated with 25 mg OCA for 72 weeks, 45% of patients in the OCA group had improved NASH activity score and reduced hepatic fibrosis, compared with 21% of patients in the placebo group. Pruritus was observed in 23% of OCA-treated patients compared with 6% in the placebo group^[84]^. In the Month 18 interim analysis of an ongoing phase III study of OCA for NASH, the histological criteria for NASH resolution endpoint was not met; however, 25 mg OCA treatment significantly improved fibrosis and key components of NASH by other criteria among patients^[85]^. Additionally, OCA worsened the circulating lipid profile, as treated patients had increased low-density lipoprotein (LDL) and decreased high-density lipoprotein (HDL) cholesterol levels^[83,84,86]^. Similar changes were also observed when OCA was administrated to healthy human subjects^[87]^. However, the adverse effects on lipid profiles could be mitigated by concomitant treatment with statins^[88]^.

Several other FXR agonists have been developed to treat NASH. Cilofexor, formerly known as GS-9674^[89]^, and TERN-101 (NCT04328077) have been tested in phase II clinical trials in NASH patients. Another FXR agonist, Tropifexor (TXR), is being tested in combination with cenicriviroc (CVC), a chemokine receptor types 2/5 antagonist, in a phase IIb trial for NASH patients^[90]^.

Furthermore, preliminary data from clinical trial also suggests that FXR activation also alters intestinal microbiota (NCT01933503). Consistent with this finding, mice fed OCA showed increased proportion of firmicutes in the small intestine^[91]^. Mouries *et al.* ^[43]^ also demonstrate the disruption of the intestinal epithelial barrier and gut vascular barrier (GVB) are early events in NASH pathogenesis. Activation of FXR by bile acid analogue or OCA drives β-catenin activation in endothelial cells, and protect against gut vascular barrier disruption and NASH development^[43]^.

### Short-chain fatty acids

Short-chain fatty acids (SCFAs) (acetate, propionate, and butyrate) are anaerobic fermentation products generated by gut microbiota from non-digestible carbohydrates such as non-starch polysaccharides, resistant starch, and miscellaneous low-digestible saccharides^[92]^. SCFAs are transferred to the liver via the portal circulation and serve as precursors for lipogenesis or gluconeogenesis^[93]^. There is evidence that SCFAs produced in the colon contribute to approximately 5%-10% of the normal daily energy requirements^[94]^.

Preclinical studies demonstrate that SCFAs activate the G-protein-coupled receptors (GPCRs) GPR41 and GPR43 of gut enteroendocrine L cells. Activation of GPCRs stimulates peptide YY (PYY) release, which slows gastric emptying and thus enhances nutrient absorption^[95,96]^. Activation of GPR41 and GPR43 in these L cells also promotes secretion of GLP-1, a peptide hormone that inhibits gastric emptying and food intake. GLP-1 also promotes hepatic lipid oxidation to reduce hepatosteatosis^[97,98]^. In addition, activation of GPR43 in adipocytes inhibits lipolysis and release of fatty acids into circulation^[99]^.

After SCFAs enter the liver via the portal vein, they undergo further metabolism. Acetate can be converted to acetyl-CoA by hepatic acetyl-coenzyme a synthetase cytoplasmic (ACSS2)^[100]^ and used as one of the major sources for *de novo* lipogenesis^[100,101]^. Propionate is a precursor for gluconeogenesis and promotes gluconeogenesis in the liver^[102,103]^. Butyrate is another precursor for hepatic *de novo* lipogenesis^[102]^. Besides functioning as metabolic substrates which feed into hepatic glucose or lipid metabolism, SCFAs can also affect hepatic metabolism by serving as signaling molecules. Propionate and butyrate activated AMP-activated protein kinase (AMPK) to increase hepatic autophagy^[104]^, a catabolic process which facilitates hydrolysis of triglyceride and releases free fatty acids for mitochondrial β-oxidation^[105,106]^. Propionate and butyrate activation of AMPK in the liver increased fatty acid oxidation and reduced HFD-induced obesity, insulin resistance, and hepatosteatosis in mice^[107,108]^. The activation of AMPK by SCFAs was mediated by increased UCP2 level and AMP:ATP ratio^[104,108]^.

SCFAs also inhibit class I and II histone deacetylases (HDACs) to modulate gene transcription. Class I and II HDACs are a group of enzymes that catalyze the removal of acetyl groups from lysine residues in histones to decrease gene transcription. Butyrate, and to a lesser degree propionate, inhibited HDACs in human colon carcinoma cells^[109]^. In bone marrow-derived macrophages, the inhibitory effect on HDACs by butyrate mediated its anti-inflammatory effect^[107]^.

Clinical studies investigated the levels of SCFAs in blood and fecal samples from patients with NAFLD. Loomba *et al.* ^[20]^ demonstrated that patients with advanced fibrosis had increased levels of acetate in their fecal samples, whereas patients with mild or moderate NAFLD had increased levels of butyrate and propionate. Michail *et al*.^[30]^ found that some SCFAs, including formate, acetate, and valerate, were decreased in children with NAFLD. Moreover, when circulating SCFAs were measured in cirrhosis patients, butyric acid level inversely correlated with inflammatory markers and serum endotoxin levels^[110]^. The reason(s) for these apparent discrepancies may be due to differences in patient age, diagnosis, diet, environmental factors, or the manner that samples were processed and measured. With regard to the latter, the determination of actual SCFA concentrations can be problematic since they are volatile substances that require immediate processing for accurate measurement. These issues notwithstanding, supplementation of SCFAs in pre-clinical models of NAFLD have demonstrated beneficial effects on NAFLD. In HFD-fed mice, supplementation of butyrate decreased inflammation in the liver and adipose tissue. Furthermore, butyrate also modified bacterial population of gut microbiota by increasing SCFA-producing bacteria and decreasing endotoxin-secreting bacteria, which in turn led to increased circulating propionate and butyrate levels and decreased endotoxin levels^[111]^. In HFD-fed mice, butyrate increased PPARα-mediated β-oxidation to reduce hepatosteatosis^[112]^. In a fat-, fructose-, and cholesterol-rich diet, butyrate supplementation also decreased serum ALT and AST levels and attenuated the progression of NAFLD in mice^[113]^. Based on these preclinical data, SCFA supplementation may exert beneficial metabolic and anti-steatotic hepatic effects. Currently, there are no published studies using SCFAs to treat patients with NAFLD.

### Ethanol

Low levels of endogenous ethanol can be generated by intestinal microbiota and thus may be involved in the development of NASH. This hypothesis is supported by the experimental evidence showing that *ob/ob* mice that developed NAFLD had higher alcohol content in the morning breath than their lean littermates; moreover, this effect was abrogated by antibiotic treatment for five days^[114]^. Intestinal ethanol production may be due to dysbiosis since some species such as *Escherichia coli* can produce significant amounts of ethanol during anaerobic conditions^[115]^. Indeed, ethanol-producing bacteria such as *Escherichia coli* and other Enterobacteriaceae were substantially increased in patients with NASH^[21]^. However, in a human study, no significant difference was identified when the ethanol in the breath of patients with biopsy-proven NASH were compared to that of healthy subjects, and only a small increase was detected in women with obesity^[116]^. These studies were limited because ethanol concentration in breath is an indirect way of measuring endogenous ethanol. When blood ethanol levels were actually measured, elevated circulating ethanol levels were observed in patients with NASH and were associated with upregulation of hepatic alcohol metabolic enzymes, such as alcohol, aldehyde dehydrogenases, and CYP2E1^[117]^. Similarly, blood ethanol levels were also significantly increased in pediatric patients with NASH^[21,118]^. In cultured hepatocytes, ethanol showed no effect on apoptosis on its own, but it sensitized hepatocytes to TGF-b-triggered apoptosis^[119]^. Besides potentially toxic effects on the liver, bacteria-produced ethanol may increase intestinal permeability and portal LPS levels to activate hepatic TLR and the inflammasome cascade to contribute to liver injury^[120]^.

### Choline and choline-related metabolites

Choline, a component of the cell membrane, is mainly obtained from red meat and eggs in the diet, although the liver can synthesize some choline endogenously^[121]^. In the liver, choline is required for the synthesis of very-low-density lipoprotein (VLDL). Thus, choline deficiency decreases synthesis and secretion of VLDL, leading to hepatic triglyceride accumulation^[122,123]^. Choline-deficient diets have been widely used in rodents to induce NASH^[124]^. Choline can be converted into trimethylamine (TMA) by intestinal microbiota, which can then be oxidized by hepatic monooxygenases to form trimethylamine N-oxide (TMAO) in the liver. TMAO is then released into the circulation^[121]^. In HFD-fed 126S6 mice, higher conversion of choline into TMA by microbiota resulted in lower bioavailability of choline^[125]^. TMAO may also act directly on the liver and contribute to the development of NAFLD. Gut microbiota convert dietary L-carnitine into TMAO, which reduced absorption of cholesterol in the gut lumen and the liver^[126]^. Dietary supplementation of TMAO also reduced bile acid synthesis enzymes and thus reduced cholesterol elimination through bile^[126]^. In support of these notions, clinical studies showed that serum levels of TMAO were higher in patients with NAFLD than in healthy subjects and were positively correlated with the level of steatosis^[127]^. Another study found that increased serum TMAO levels were significantly associated with NASH in patients with T2DM^[128]^. However, it is not known whether serum TMAO serves as a biomarker for NAFLD or other types of metabolic conditions^[129]^.

### Ammonia

Inability to generate urea from amino acids during end-stage liver disease, particularly during NASH and cirrhosis, can lead to hyperammonia. When severe, patients can develop hepatic encephalopathy (HE). Hyperammonia thus can be a marker to measure the severity of liver disease^[130]^. During NASH, ornithine transcarbamylase (OTC) and carbamoylphosphate synthetase (CPS1) mRNA, protein, and activity were reduced, leading to increased ammonia concentration^[131]^. Interestingly, steatosis in primary hepatic cells was also associated with OTC and CPS1 promoter hypermethylation, decreased OTC and CPS1 gene expression, and ammonia generation^[131]^. Moreover, ammonia itself had direct effects on hepatic stellate cells (HSCs) by activating them in cell culture and *in vivo* ^[132]^. These findings suggested that the hyperammonemia that occurred during NASH and cirrhosis may itself contribute to the progression of fibrosis.

Ammonia also is generated from amino acids in the gut by Gram-negative anaerobes, clostridia, enterobacteria, and *Bacillus* spp.; Gram-positive non-sporing anaerobes, streptococci, and micrococci; and lactobacilli and yeasts that produce small amounts of ammonia^[133]^. Thus, the composition of the gut microbiome also contributes to circulating ammonia levels. However, the precise amount of ammonia generated by gut microbiota and their role in determining serum ammonia levels during NASH and cirrhosis is not well understood. Additionally, it is possible that endotoxin and inflammation by gut flora may contribute to increased uptake of ammonia from gut into the bloodstream and thereby contribute to the latter’s toxic effect on the liver^[132]^.

## Treatment Strategy for Nash Targeting Gut Microbiome

The current recommended treatment for NAFLD patients is lifestyle modification, which includes, exercise, diet, and weight loss to correct of underlying risk factors such as obesity and diabetes. Pharmacologic treatments to improve insulin sensitivity, reduce oxidative stress and inflammation, or downregulate fibrosis mechanisms have been proposed for NAFLD but are not proven. There is also evidence emerging that suggests a potential role for altering gut microbiota to treat patients with NAFLD and NASH [Table 3]. Thus far, several approaches to alter the gut microbiota have been tested in NAFLD patients, including antibiotics; supplementation of pro-, pre-, or synbiotics; and fecal transplantation^[134]^.

### Antibiotics

Several studies have examined the effect of antibiotics on NAFLD. In a preclinical model, NAFLD improved after the administration of an antibiotic cocktail (bacitracin, neomycin, and streptomycin)^[135]^. In addition to suppressing local or systemic infections, antibiotics may also regulate inflammation caused by intestinal microbiota. In this connection, treatment with cidomycin increased small intestinal transit rate and also reduced the serum levels of ALT, AST, and TNF-α in NASH^[136]^. Gangarapu *et al.* ^[137]^ also showed that rifaximin treatment significantly reduced proinflammatory cytokines, ALT, and NAFLD-liver fat score. This improvement by antibiotics was attributed to alterations in the gut microbiota population and bile acid metabolism as well as to reduced FXR signaling and decreased ceramide levels in the liver. Despite these potential beneficial effects, antibiotics need to be used judiciously and may not be appropriate therapy for most patients since they will reduce normal bacterial flora and increase the risk for overgrowth by pathogenic bacteria such as *Clostridium difficile.*

### Prebiotics, probiotics, and synbiotics

There are numerous ongoing studies investigating the feasibility of using pre-, pro-, and synbiotics as therapeutic strategies for NAFLD/NASH. Prebiotics are indigestible food products that do not contain any living organisms but are able to promote the growth and metabolism of bacteria that ferment prebiotics to SCFAs. Probiotics are defined as viable bacteria which, upon ingestion, help improve intestinal mucosal integrity by modulating the gut microbiota to confer health benefits to the host. The combined use of pre-and probiotic approaches is called synbiotic (or symbiotic) therapy^[138,139]^.

Prebiotics might be ideal treatment candidates for NAFLD due to their low cost and safety profile; however, their effectiveness remains unproven. Prebiotic treatment of NASH patients with oligofructose 16 g/d showed significant reduction in serum aspartate aminotransferase level; however, the same cohort had no significant difference in serum triglyceride levels when compared to placebo after eight weeks of treatment^[140]^. A recent meta-analysis of histologically confirmed NAFLD patients showed that use of prebiotics caused a modest reduction in serum ALT and AST but only a very small reduction in BMI. In the same cohort, no changes in serum inflammatory markers and total cholesterol were observed. In another study, prebiotics reduced low-density lipoprotein cholesterol (LDL-c) and HDL^[141]^. However, a systematic review of clinical studies on the use of prebiotics for obesity-induced NASH did not encourage the usage of prebiotics due to lack of quality studies^[140]^. Additionally, an important consideration is that different prebiotics may have distinct effects on the liver and variable effects in different individuals. Thus, the efficacy of prebiotics as a treatment for NAFLD is currently still unresolved.

Serum markers of enzymatic dysregulation or biomarkers for liver injury have been used to evaluate the efficacy of probiotics. A recent meta-analysis showed that the use of probiotics significantly reduced liver transaminase, TNF, and insulin resistance^[142]^. Probiotics have also been used in combination with *Lactobacillus* and Bifidobacteria species^[143]^. However, a systematic review highlighting the three clinical studies which examined the efficacy of probiotics in patients with NAFLD thus far did not support their usage in NASH due to lack of high-quality studies^[142]^. Nonetheless, more recent studies showed promise for this approach. Alisi *et al*.^[144]^ showed that obese children who received probiotics composed of a mixture of eight strains daily for four months had a significantly lower risk for severe steatosis compared to placebo. Liu *et al*.^[145]^ showed that probiotic culture supernatant improved metabolic function by activating the FGF21/adiponectin pathway in a preclinical model. They showed that *Lactobacillus rhamnosus* culture supernatants (LGGs) reduced NASH generated by high fat/high fructose diet plus intermittent hypoxia exposure (HFDIH). The authors showed that treatment with LGGs increased hepatic FGF21 mRNA expression and circulating FGF21 protein levels, as well as increased hepatic PPARα expression and fecal butyrate concentration. Further pre-clinical and clinical studies of probiotics are needed to determine their potential efficacy in NAFLD^[145]^.

Synbiotics have been used to treat NAFLD in animal studies and adult patients. Raso *et al*.^[146]^ showed that rodents with HFD had improvement in inflammation and decreased amounts of *Enterobacteriales* and *Escherichia coli* in colonic mucosa when given synbiotics. Malaguarnera *et al*.^[147]^ assessed 66 histologically diagnosed NASH patients receiving synbiotics for 24 weeks and found significant reduction in the TNF-α and C-reactive protein (CRP) levels as well as histological improvements when compared to normal control patients. In a large placebo-controlled trial (N = 80), ultrasound-diagnosed NAFLD patients who received synbiotics for eight weeks had significant reductions in steatosis compared to their baseline levels whereas patients who received placebo displayed no improvement. However, no significant differences in CRP, ALT, and AST levels were observed between the two groups^[148]^.

When taken together, preliminary evidence suggests pre-, pro-, and synbiotic treatments for NAFLD may potentially provide clinical benefits to patients. Thus far, no severe adverse effects have been reported during any randomized control trials (RCTs), which also broadens their potential application. There are still some ongoing clinical trials (NCT03585413 and NCT01791959) examining the role of these compounds in NAFLD. Notably, the beneficial effects of pre-, pro-, and synbiotics may vary among individuals, owing to differences in dietary habits, intestinal microflora, and genetic backgrounds of the hosts. This heterogeneity introduces variability in individual responses. However, it is possible that individualizing treatments based on microbiota composition in stool and other patient considerations could improve results. Thus, optimizing the dose and determining the most effective type of therapy may be necessary for each individual.

### Fecal microbiota transplantation

Fecal Microbiota Transplantation **(**FMT) is a new treatment approach to repopulate gut microbiota of patients with healthy intestinal flora. FMT has been successfully used in treating patients with refractory and recurrent *Clostridium difficile* and for other diseases by increasing the microbiome diversity^[149,150]^. It is an exciting new approach that is being actively explored as a treatment for NAFLD. Studies from Zhou *et al*.^[151]^ and Le Roy *et al*.^[13]^ suggested that FMT attenuated HFD-induced NASH in mice by improving intrahepatic lipid accumulation, IR, and serum proinflammatory cytokine levels. Another study showed that animals with NAFLD which underwent fecal transplant had decreased hepatic gluconeogenesis and intestinal permeability^[152]^. In a RCT, patients with metabolic syndrome who received gut microbiota from healthy individuals had increased insulin sensitivity and gut microbial diversity six weeks after FMT^[153]^. Gracia-Lezana *et al.* ^[154]^ also showed that restoration of gut microbiota normalized portal hypertension in rodent models of NASH.

Thus far, these early studies suggest that FMT might have positive effects on NAFLD/NASH. The growing interest in FMT as a potential treatment approach for NAFLD/NASH has led to several ongoing clinical trials (NCT03803540, NCT02469272, NCT02721264, NCT02496390, and NCT02970877). However, more high quality studies are needed to determine the efficacy and safety of FMT for NAFLD. Additionally, optimization of FMT protocols, bacterial species to be transplanted, sample preparation, and dosage need to be determined.

## Gut Microbiota and Nash-Associated HCC

The transition from NAFLD to NASH involves steatosis, lobular inflammation, and progressive liver fibrosis. Although NAFLD may represent benign accumulation of liver fat alone, NASH is marked by lipotoxicity that promotes the activation of intracellular stress kinases and apoptosis^[54]^, leading to both inflammation and concomitant fibrosis^[155]^. The development of advanced NASH is worrisome clinically since some patients with this condition not only progress to cirrhosis but also have increased risk for hepatocellular carcinoma (HCC)^[156]^. The first documentation of HCC associated with NASH was reported by Powell *et al*.^[157]^ in their five-year follow-up study of 42 patients with NASH. Another follow-up study showed that 2.6% of patients with NASH developed cirrhosis compared with 4% of patients with HCV^[158]^. A seminal study by Sanyal *et al*.^[159]^ identified NAFLD/NASH as the most common underlying risk factor for HCC in the U.S., as it was present in 59% of cases. Corroborating studies performed in Asia and Europe also have supported these findings^[160,161]^.

Animal models of NAFLD/NASH also show similar propensity to develop HCC^[162]^. Multiple hits in the form of genetic predisposition, epigenetic modifications, immunological surveillance, endocrine defects, and gut microbiome mediate the transition from NAFLD to HCC^[163]^. Hepatocyte apoptosis is also a factor that promotes HCC^[164]^. Detailed molecular pathogenesis of NASH-associated HCC is reviewed elsewhere**[163,165,166]**.

The gut microbiome plays a significant role in the progression of NASH to HCC^[167,168]^. NASH-associated dysbiosis and increased intestinal permeability cause the release of pathogen-associated molecular patterns (PAMPs) and gut microbiome-derived metabolites to increase liver inflammation and lipogenesis^[23]^. At the molecular level, PAMPs activate TLR-induced cytokine and chemokine production (e.g., IL8, IL-17, and IL1β) to increase immune cell infiltration of the liver^[169]^. Increased cytokine surge also leads to elevated oxidative stress and DNA damage, which could then cause HCC initiation^[170]^. PAMPs and another microbiota-derived metabolite, lipoteichoic acid (LTA), also activate hepatic stellate cells (HSCs) via senescence-associated secretory phenotype (SASP) and promote hepatocyte proliferation and increase predisposition to HCC^[169,171]^.

Bile acid metabolism also plays an important role in NASH to HCC transformation. An association between altered bile acid metabolism and HCC has been documented in both human and preclinical animal studies^[172,173]^. High levels of bile acids in the liver can induce hepatocyte DNA damage, apoptosis, and inflammation, thus promoting tumorigenesis^[174,175]^. Additionally, dysbiosis in NASH leading to increased abundance of Gram-positive gut microbiota promotes HCC by augmenting the production of secondary bile acids such as deoxycholic acid (DCA), inhibiting liver sinusoidal endothelial cell (LSEC) activation and leading to chemokine ligand 6 (CXCL6) suppression, natural killer T cell recruitment, and carcinogenesis^[176]^. Furthermore, secondary BAs also directly contribute to HCC development by activating mTOR signaling^[177]^. Thus, controlling BA metabolism via antibiotics could potentially lead to prevention of HCC development^[177]^.

## Conclusion

The development and progression of NAFLD is a complex and multifactorial process that involves genetic and environmental/host effects. Additionally, most NAFLD patients have co-morbidities associated with metabolic syndrome, which also impact the liver. Pre-clinical and clinical evidence suggests that altered gut microbiota, particularly the overgrowth in the small intestine and changes in the composition of microbiota, likely contribute to NAFLD progression. Although there are some general consistent changes in the gut microbiome flora, distinct gut microbiota composition can be found in different individuals, suggesting many different types of bacteria may be involved. Although gut microbiota can affect intestinal permeability, fewer than half of patients with NAFLD exhibited increased intestinal permeability, suggesting that gut metabolites or intestinal inflammation have primary effects on the liver. A few microbiota-related metabolites have been identified thus far that are positively- or negatively-associated with NAFLD progression [Table 2], although the number of identified metabolites with microbial origin currently is limited. Monitoring different metabolites as non-invasive biomarkers related to changes in the gut microbiome and NAFLD progression in stool, serum/plasma, and urine may offer the possibility for better diagnosis and personalized treatment. Understanding of the role of microbiota in NAFLD is still in its infancy. However, the identification of microbiota signatures and therapeutic modification of the microbiome provide new possibilities for the diagnosis and treatment of NAFLD.

## Figures and Tables

**Table 1:** Changes of microbiota in NAFLD patients

Disease	Phylum	Family	Genus	Species
NAFLD	Proteobacteria^[16,20]^	Enterobacteriaceae^[17,20]^	Escherichia^[16,20]^	
		Rikenellaceae^[18,21]^	Dorea^[18,22]^	
		Rumminoccaceae^[17,20]^	Anaerosporobacter^[21,23]^	
			Coprococcus^[16,20,21]^	
			Eubacterium^[16,20]^	
			Faecalibacterium^[20,23]^	
			Prevotella^[16,24]^	
Liver fibrosis or cirrhosis	Proteobacteria^[27]^	Enterobacteriaceae^[26]^	Streptococcus^[26,27]^	Escherichia coli^[19]^
	Fusobacteria^[27]^		Gallibacterium^[27]^	Bacteroides vulgatus^[19]^
	Bacteroidetes^[27]^			Eubacterium rectale^[19]^
Children with NASH				Prevotella copri^[25]^

NAFLD: non-alcoholic fatty liver; NASH: non-alcoholic fatty liver

**Table 2:** The effects of microbiome products on the liver

Microbiome products	Effects on Liver
Endotoxins, Bacterial DNA, Bacterial peptide glycans	Increase gut permeability, gut inflammation, liver steatosis, inflammation, and fibrosis
Altered bile metabolites	Increases liver bile acids conversion to unconjugated and dehydroxylated secondary bile acids (e.g., deoxycholic and lithocholic acids). Activates FXR to repress bile acid synthesis, regulate lipogenesis, and decrease fibrosis
Short-chain fatty acids (acetate. propionate, butyrate)	Stimulate gut endocrine cells to secret GLP-1 to increase hepatic fatty acid β-oxidation. Decrease endotoxin-producing bacteria. Acetate and butyrate can serve as precursors for hepatic lipogenesis; however, they also can increase PPARα-mediated β-oxidation of fatty acids and decrease hepatosteatosis
Ethanol	Increases gut permeability and LPS-mediated inflammation in liver. Potential direct toxic effects on liver
Choline/Choline-related metabolites	Convert choline into TMA and then oxidized into trimethylamine N-oxide (TMAO) in the liver. TMAO inhibits cholesterol conversion into bile acids. Associated with hepatosteatosis
Ammonia	May increase hepatosteatosis and hepatic stellate cell activation

**Table 3:** Clinical trials modulating microbiome or its metabolites for NAFLD patients

Intervention	Potential agent	NCT number	Targeted conditions	Phase
FXR agonist	obeticholic acid	NCT02548351	NASH	Phase 3
FXR agonist	Cilofexor	NCT03449446	NASH	Phase 2
FXR agonist	TERN-101	NCT04328077	NASH	Phase 2
FXR agonist in combination with CCR2/5 antagonist	Tropifexor in combination with cenicriviroc	NCT03517540	NASH	Phase 2
Synbiotic		NCT01791959	NASH	Phase 2
Antibiotic	Solithromycin	NCT02510599	NASH	Phase 2
Probiotics		NCT03585413	NAFLD	Phase 3
FMT		NCT02496390	NAFLD	Phase 2
FMT		NCT02970877	NAFLD	Phase 2

FXR: farnesoid X receptor; FMT: Fecal Microbiota Transplantation; NASH: non-alcoholic steatohepatitis; NAFLD: non-alcoholic fatty liver

## Data Availability

Not applicable.
